# Lower levels of leptin are associated with severity parameters in visceral leishmaniasis patients

**DOI:** 10.1371/journal.pone.0214413

**Published:** 2019-03-26

**Authors:** Aline Mireille da Cunha Fievez, Maria Luciana Silva-Freitas, Anastácio de Queiroz Sousa, Joanna R. Santos-Oliveira, Alda M. Da-Cruz

**Affiliations:** 1 Hospital São José de Doenças Infecciosas, Universidade Federal do Ceará, Fortaleza, Brazil; 2 Laboratório Interdisciplinar de Pesquisas Médicas–Instituto Oswaldo Cruz—FIOCRUZ, Rio de Janeiro, Brazil; 3 Núcleo de Ciências Biomédicas Aplicadas, Instituto Federal de Educação, Ciência e Tecnologia–IFRJ, Rio de Janeiro, Brazil; 4 National Institute of Science and Technology on Neuroimmunomodulation (INCT-NIM), Rio de Janeiro, Brazil; Academic Medical Centre, NETHERLANDS

## Abstract

Visceral leishmaniasis (VL) is the most severe clinical form of leishmaniasis, and if untreated may be fatal. It affects important organs of the immune system and is characterized by a specific immunosuppression, along with intense cellular activation and cytokine storm. Moreover, VL is now recognized as a systemic inflammatory response syndrome (SIRS), in which multiple cytokines and other pro-inflammatory molecules are released. The action of these inflammatory mediators may be considered risk factors for poor prognosis and death. Leptin, a hormone derived from adipose tissue, has been described with several immunoregulatory functions *in vitro* and *in vivo Leishmania* infection models, particularly for enhancing the macrophage microbicidal mechanisms. Considering that evaluation of immunologic parameters that may be associated with this clinical scenario may help to decrease VL lethality, we evaluated whether leptin is associated with VL pathogenesis. Thirty-one patients were recruited in the active phase of VL, of which 22 were followed up until one month after therapy (1mpt). Except for creatinine levels, all clinical parameters were altered in active VL patients, especially leucocyte counts and albumin and hemoglobin levels. Also, elevated levels of lipopolysaccharide (LPS), immunoglobulins (Ig)G1 and G3 anti-*Leishmania* and interleukins (IL)-6 and -10 were higher than in healthy individuals. In contrast, active VL patients presented diminished serum leptin levels and positive correlation with leukocytes counts and hemoglobin and albumin levels. After 1mpt, VL patients showed a significant increase in leptin levels, reaching values similar to healthy volunteers. As expected, only LPS levels remained elevated after 1mpt. These findings suggest that leptin levels are affected in *Leishmania* infection and the correlation with important parameters associated with the prognosis of VL points to the involvement of this molecule in VL immunopathogenesis. Additional studies are needed to evaluate the possibility of leptin as a prognostic marker of VL.

## 1. Introduction

Visceral leishmaniasis (VL), also known as kala-azar, is the most severe clinical form of leishmaniasis due to frequent complications and if untreated, increases the risk of death. It is present in geographic areas with conditions of poverty, which contribute to the continuity of social inequality. Actually, VL is endemic in 98 countries and around 200,000–400,000 new cases are diagnosed every year. In the Americas, the majority of the cases are concentrated in Brazil, which reported 4,103 cases in 2017 with a lethality rate reaching around 8.8% in the last 10 years [1]. Among these cases, the Northeast region remains the first Brazilian region in number of notifications, whose Ceará state records the second highest number of cases.

Some factors inherent to the parasite (strain, virulence) and host factors (genetic, nutritional status, age and immune response) will dictate the clinical outcome of the disease, which can be presented as asymptomatic, classic, or severe. Typically, classical VL is characterized by anemia, fever, and hepatosplenomegaly along with nutritional deficiency and weight loss. However, for reasons not yet fully understood but that should include the parasite and the effector immune responses, some patients may progress to the more severe forms of the disease, which can be fatal in some cases. In this context, the decrease of VL lethality rate should take into account the physician’s knowledge about signs of disease severity but also the evaluation of immunological parameters that may be associated with this clinical scenario.

VL affects important organs of the immune system, which in turn may compromise the effector immune responses and therefore, leading to a specific immunosuppression in response to the parasite [2]. On the other hand, despite the impairment of the specific response, an intense degree of cellular activation is observed. Active VL is now understood as a severe systemic inflammatory syndrome [3], in which elevated levels of interleukin (IL)-10 and inflammatory cytokines (IL-6, -8, -17, interferon [IFN]-γ, macrophage migration inhibitory factor [MIF], tumor necrosis factor [TNF]) [4–8], microbial products as lipopolysaccharide (LPS) [7], and soluble factors such as soluble CD14 [9], prostaglandin F2α, leukotriene B4, resolvin D1 [10], neopterin [11], and sCD163 [12] are associated with worsening of patients' clinical status. Thereby, the clinical and systemic actions of these inflammatory mediators may be considered risk factors related to poor prognosis and death.

After treatment, the spleen or liver size presents a steady decrease but returns to normal values at only 120 days [4]. In this context, VL patients present a gradual reduction of inflammatory mediator levels (IL-6, IL-8 and IL-10) starting at 30 days after infection [10]. However, a delay in the effector immune response is observed since several soluble factors have not yet returned to normal even after six months of specific treatment [7]. Also, a decrease of IgG1 and IgG3-specific levels in response to *Leishmania* after six months of therapy may be a useful biomarker for monitoring the post-therapeutic cure in human VL [13,14]. More studies on laboratory parameters in order to predict successful therapeutic responses are needed and could help to elucidate the steps of the immune response’s refreshment toward homeostatic status.

Leptin, a protein described in 1934 in studies on obese and diabetic mice, presents a multifactorial role in the immune system [15]. It is produced and secreted mostly by adipocytes, so that a positive correlation between body mass index (BMI) and circulating levels of leptin is observed in humans. Leptin may act as a hormone to regulate the phagocytic function and secretion of inflammatory cytokines in addition to increasing reactive oxygen species (ROS) production, granulocytes chemotaxis, and enhancement of Th1-associated immune responses. Earlier studies have already described that leptin or leptin receptor deficiency can contribute to an increase in susceptibility to bacterial infections and pneumonia [16]. *In vitro* studies have shown that in the presence of recombinant human leptin, *L*. *donovani-*infected macrophages presented an enhancement in phagocytic activity and IL-1α, -1β, -8 and TNF production [17]. Due to the crucial role of macrophages in the clearance of amastigotes, we hypothesized that weight loss in VL, either due to previous malnutrition or even during the course of the disease could lead to reduction of the leptin levels, which in turn could impair microbicidal functions.

In this context, the purpose of this study was to evaluate whether leptin is associated with VL pathogenesis. We demonstrated that the leptin levels were reduced during the active phase of infection and correlated with laboratory markers of severity but returned to normal levels after therapy.

## 2. Materials and methods

### 2.1 Study design and participants and ethical aspects

Thirty-one individuals with VL were evaluated from May 2015 to August 2016. They were recruited from the São José Hospital of Infectious Diseases at Fortaleza, Ceara, Brazil. VL diagnosis was based on the presence of specific antibodies to the recombinant k39 antigen using a commercial anti-k39 rapid test (Bio-Rad, Rio de Janeiro, Brazil) as recommended by the Ministry of Health of Brazil. At the time of admission to the study, all patients presented characteristics that are considered risk factors for severe VL according to the Brazilian Ministry of Health, which justified their hospitalization for treatment [18]. Several parameters for inclusion were monitored: (1) leukocyte counts <1000 cells/mm^3^; (2) platelet counts <50,000/mm^3^; (3) hemoglobin levels <7.0 g/dL; (4) albumin levels <2.5 g/dL; and (5) fever for >60 days [18]. Peripheral blood was then collected from all patients for the hematological and laboratory analyses and was characterized as the active phase of VL based on the above-described parameters. Twenty-two patients underwent clinical follow-ups one month after the end of the anti-*Leishmania* treatment (1 mpt). During this visit, it was possible to obtain biological samples of peripheral blood to perform clinical and laboratory assessments. Exclusion criteria included several parameters: (1) individuals who had relapses of VL; (2) pregnant women; (3) individuals with autoimmune diseases; and (4) immunosuppression due to diseases or use of immunosuppressive drugs.

A group of ten individuals from the same endemic area and without history of VL were included as controls for the laboratory analyses, which included LPS levels, specific anti-*Leishmania* antibodies, leptin, and inflammatory cytokines. They came from the Center of Hemotherapy and Hematology of the Ceara State (HEMOCE).

This study was approved by Research Ethics Committee of the São José Hospital of Infectious Diseases and the Oswaldo Cruz Foundation under protocol number 922.417. Individuals who met the inclusion criteria were invited to participate in the study and signed the informed consent term.

### 2.2 Assessment of lipopolysaccharide (LPS)

LPS levels were quantified using a commercial assay kit (Limulus Amebocyte Lysate [LAL] QCL-1000; Cambrex, Milan, Italy). Each series of determinations included a blank and four endotoxin standards run in duplicate. The samples were diluted in endotoxin-free water (1:2), and 50 μl of sample or standard were dispensed into the appropriate microplate. Then, the LAL followed by the substrate solution were added to produce a colorimetric reaction. The absorbance was determined at 405–410 nm via spectrophotometry. The results are expressed as picograms per milliliter (pg/mL), and the sensitivity level was 10 pg/mL.

### 2.3 Anti-*Leishmania* immunoglobulin detection

An enzyme-linked immunosorbent assay (ELISA) was performed as described in detail previously by Fagundes-Silva et al (2012) [19], but some modifications were necessary. Briefly, *L*. *(L*.*) infantum* (MHOM/BR/1974/PP75) promastigote soluble antigens (40 μg/mL) were used to coat the wells of a polystyrene flat-bottom microtiter plate (Nunc-Immuno, Roskilde, Denmark). Besides this, diluted peroxidase-conjugated mouse monoclonal anti-human antibodies, such as IgG (1:1000) (Invitrogen, San Francisco, CA, USA), IgG1 (1:200) and IgG3 (1:400) (Zymed Laboratories Inc., San Francisco, CA, USA) were used. The absorbance was measured at 492 nm using a microplate reader (Benchmark, Bio-Rad Laboratories, Hercules, CA, USA). The results were expressed as ELISA index (EI).

### 2.4 Quantification of serum Leptin and IL-6 levels

Leptin and IL-6 levels were determined using an ELISA with commercial kits (Human Leptin and IL-6 Quantikine ELISA, respectively, R&D Systems, Minneapolis, MD, USA). The assay diluent was added to all wells of the 96-well microplate provided by kit, and then 100 μL of samples were added per well. Also, a standard curve of seven-points diluted in calibration diluent, and a blank was performed in duplicate. The human leptin or IL-6 conjugate was added to each well and incubated for 1 h. The colorimetric reaction occurred after the substrate solution (3,3′,5,5′- tetramethylbenzidine) was added to the microplate. The optical density was determined at a wavelength of 450 nm by spectrophotometry, and the results were expressed as picograms per milliliter (pg/mL).

### 2.5 Assessment of IL-10 in the serum

The levels of IL-10 was determined using an ELISA using the commercial kit Duoset (R&D Systems, Minneapolis, MD, USA). The human detection antibody (anti-IL-10) was diluted in accordance to the protocol, and incubated for 2 h. The colorimetric reaction occurred after streptavidin-HRP followed by the substrate solution (3,3′,5,5′- tetramethylbenzidine) were added to the microplate. The reading was performed in a spectrometer at a wavelength of 450 nm, and the results were expressed as picograms per milliliter (pg/mL).

### 2.6 Statistical analyses

Continuous variables were expressed as median and interquartile range (IQR). Statistical analyses were performed by a non-parametric analysis using the Mann-Whitney test when two groups were compared. Analysis of variance (ANOVA) Kruskal-Wallis test followed by Dunns’ test was used when three groups were compared concurrently. The Spearman’s test was used for correlation analysis. The statistical analyses were performed using GraphPad Prism software (version 6.0, San Diego, CA, USA). Differences were considered statistically significant when the p value was <0.05.

## 3. Results

### 3.1 Clinical characteristics and severity parameters in the active phase of VL

Thirty-one VL patients were studied in the active phase, and 22 were prospectively followed up until one month after anti-*Leishmania* therapy. The clinical parameters of VL patients are shown in Table 1. The majority of them were male (93.5%), the median age was 42 years old (IQR: 33–52), and the median weight was 68 kg (IQR: 57.8–77.8 kg). The patients did not evolve to severe VL and none of them presented clinical signs of bacterial infection. Except for creatinine levels, the laboratory parameters were outside the reference values but the prospectively evaluated patients showed values within normal ranges after one-month post-treatment. It is important to stress that total regression may take a few months, but some parameters improved after the second week.

**Table 1:** Clinical characteristics of the visceral leishmaniasis patients in the active phase of disease.

Patient ID	Age	Sex	Weight (Kg)	Leukocytes (cells/μL)	Neutrophils (cells/μL)	Lymphocytes (cells/μL)	Hemoglobin (g/dL)	Platelets (/μL)	Creatinine (mg/dL)	AST (U/L)	ALT (U/L)	Albumin (g/dL)
**P01**	54	M	74	4760	2903	1142	7.4	81000	1.8	22	13	3.8
**P09**	56	M	70	2640	1135	1214	9	140000	0.9	81	105	3.6
**P10**	54	M	60	3910	1173	2189	11.3	77000	0.7	77	33	3.6
**P11**	39	M	—	6560	3083	3017	11.2	87000	1	154	181	4.8
P13*	24	M	—	2380	1237	833	8.1	78000	0.8	261	135	2.4
**P17**	31	M	68	2000	600	1180	6.9	67000	0.8	78	65	2.7
**P18**	41	F	69.8	2010	1105	763	8.8	66000	0.5	75	28	3.0
**P22**	32	M	64	1350	567	594	7	61000	1	116	33	2.6
**P23**	44	M	45	960	201	672	6.2	29000	0.6	123	50	1.2
**P30**	34	M	79	1970	847	906	7.8	117000	1.1	33	12	3.6
**P37**	46	M	82	1160	777	290	5.8	54000	0.8	93	53	3.4
P38*	33	M	67.6	2180	1242	697	9.4	113000	0.9	848	1088	3.3
**P45**	56	M	68	1480	790	399	9	74000	0.7	—	12	3.9
P46*	47	M	56	1380	540	560	7.9	173000	0.6	—	22	3.3
**P48**	44	M	56	2620	1362	786	9.2	154000	0.8	54	94	3.6
**P49**	43	M	130	3090	1792	1041	12.7	55000	0.8	85	113	3.5
**P52**	42	M	65	4630	1805	2129	7.8	466000	0.8	—	54	—
P56*	43	M	48	1980	1360	409	7.8	76000	1.2	—	40	—
**P58**	22	M	89	1490	600	745	8.5	102000	3.3	57	39	3.4
**P61**	52	M	88	2110	1329	611	9.8	61000	0.9	87	64	3.9
**P64**	24	M	—	3280	1541	1410	10.8	65000	1.1	29	23	4.3
**P68**	33	M	—	3720	1748	1674	11.2	218000	1.0	133	69	3.2
**P69**	57	M	68	2370	1990	284	7.5	23000	1.0	36	46	3.6
**P76**	57	M	—	4220	2489	928	7.4	86000	1.7	102	62	2.2
P77*	35	M	—	3500	910	2485	9.8	153000	1.2	250	418	3.6
**P82**	47	M	72	1150	575	425	6.7	60000	1.4	13	13	3.3
P88*	19	F	—	2630	1393	1180	8.7	13000	1.5	—	108	2.5
P89*	39	M	68	2110	527	1498	6.7	141000	0.9	—	19	3.0
P93*	40	M	57	3730	2461	783	12.0	134000	0.9	23	14	3.2
P102*	55	M	54.4	1170	713	351	7.2	54000	3.0	42	33	2.7
**P103**	32	M	83	2730	1965	546	10.4	118000	0.9	260	49	4.0
**Median (IQR)**	42 (33–52)	NA	68 (57.8–77.8)	2370 (1490–3500)	1237 (713–1792)	786 (560–1214)	8.5 (7.4–9.8)	78,000 (61,000–134,000)	0.9 (0.8–1.2)	81 (39–128)	49 (23–94)	3.40 (2.85–3.60)
***Reference values***	*NA*	*NA*	*NA*	*3900–10700*	*1600–7500*	*800–4500*	*F*: *12–16* *M*: *14–17*	*150*,*000–350*,*000*	*0*.*7–1*.*3*	*0–35*	*0–35*	*3*.*5–5*.*5*

* refers to the patients who did not complete the clinical follow up until 1 month post treatment.

NA: not applicated; F: female; M: male; IQR: interquartile range.

### 3.2 High levels of lipopolysaccharide (LPS) were observed in the active and post-treatment phases in VL patients

**Fig 1:**
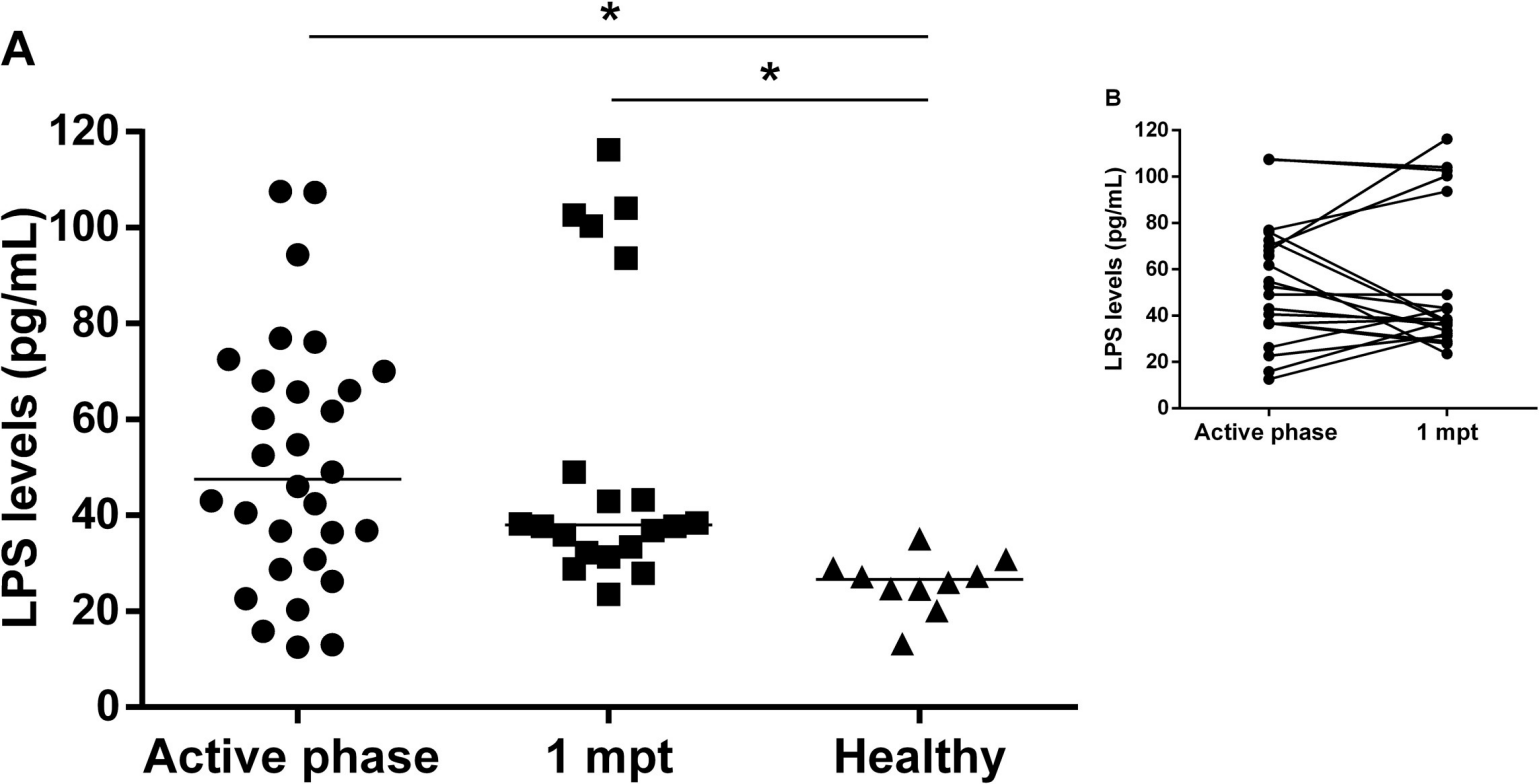
Plasma LPS levels in visceral leishmaniasis (VL) patients during clinical follow up. **(A)** LPS levels were analyzed in the active phase and one-month post-treatment (1 mpt) in VL patients and in healthy volunteers. **(B)** The insert shows the LPS levels from the same patients prospectively evaluated in two different time points (n = 22). Each symbol represents one patient. The horizontal bar represents the median values. Asterisks denote a statistically significant difference between groups, *p<0.05; **p<0.01.

Considering that LPS may be implicated in VL pathogenesis as an important co-factor for increasing cellular level activation, we assessed the LPS levels in the active phase (Fig 1A) and for those who were followed-up until the end of treatment (one month post treatment, 1mpt) (Fig 1B).

Active VL patients presented significantly elevated LPS levels (median: 47.6 pg/mL, IQR: 30.4–68.6 pg/mL) when compared to healthy volunteers (median: 26.6 pg/mL, IQR: 23.5–35.2 pg/mL) (Fig 1A). Despite the reduction of LPS 1mpt (median: 38 pg/mL, IQR: 32.5–82.5 pg/mL), the differences were not statistically significant in comparison to the active phase of VL, but they were still higher than levels in healthy controls (p <0.05). Remarkably, twelve out of 20 VL patients prospectively followed-up maintained or even presented augmented the LPS levels 1mpt, suggesting that co-factors related with cellular activation status can still be biologically active (Fig 1B).

### 3.3 Reduction of anti-*Leishmania* IgG1 and IgG3 and also IL-6 and -10 cytokines post-treatment reinforces their role as laboratory biomarkers in clinical management of VL

**Fig 2:**
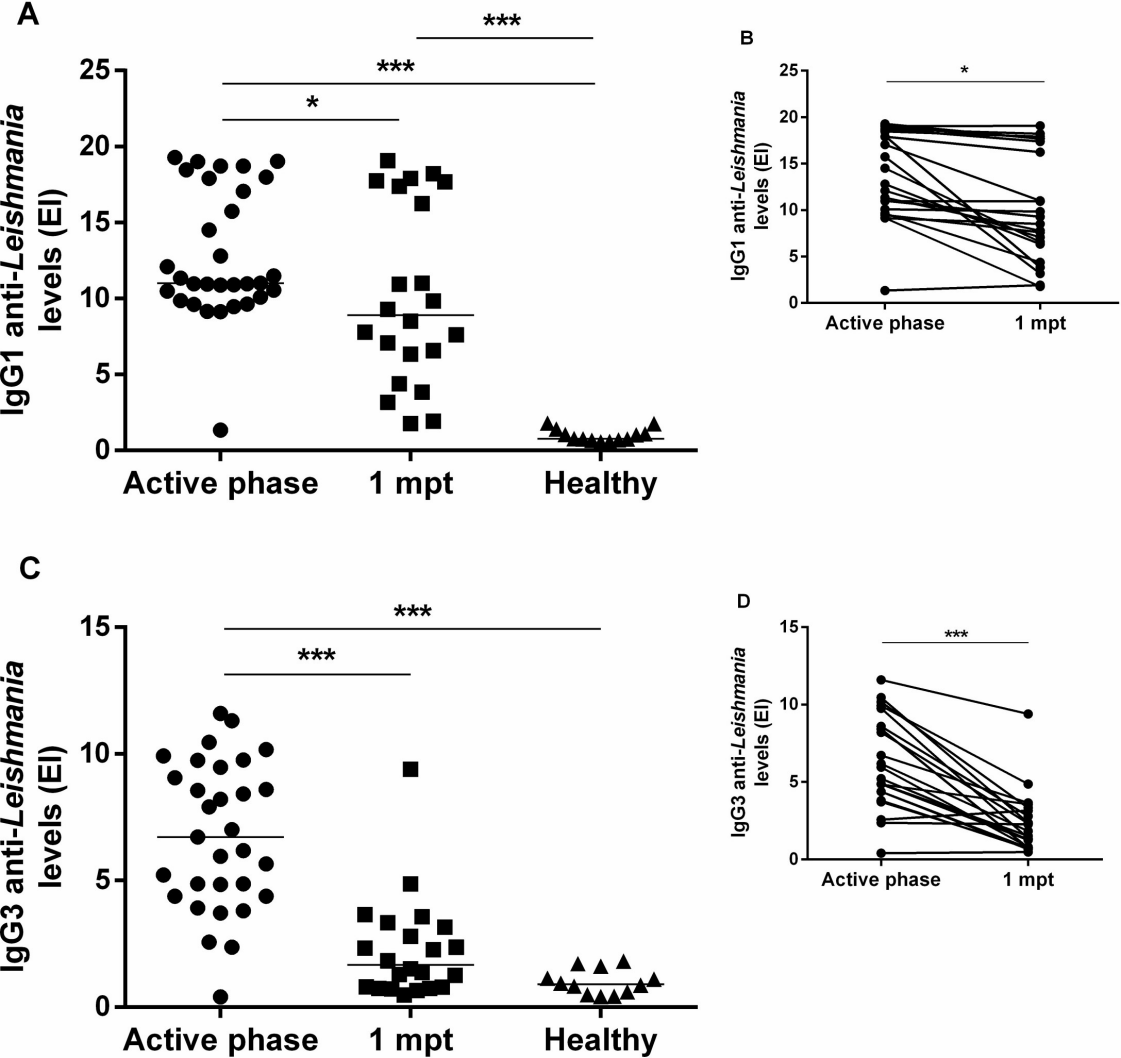
Titers of the anti-*Leishmania (L*.*) infantum* immunoglobulin G1 (IgG1) and IgG3 in visceral leishmaniasis (VL) patients during clinical follow up. **(A and C)** IgG1 and IgG3 levels of VL patients in the active phase, after one-month post-treatment (1 mpt) and in healthy volunteers. **(B and D)** The insert shows the IgG1 and IgG3 levels from the same patients prospectively evaluated in two different time points (n = 22). Each symbol represents one patient. The horizontal bar represents the median values. Asterisks denote a statistically significant difference between groups, *p<0.05; ***p<0.0001.

As expected, anti-*Leishmania* IgG1 (median: 11.0; IQR: 10.0–17.9) and IgG3 (median: 6.7; IQR: 4.4–9.5) were positive in the active phase of the disease (Fig 2A–2D). The levels of both IgG1 (median: 8.9; IQR: 5.9–17.5) and IgG3 (median: 1.7; IQR: 0.8–3.2) had a reduction 1mpt in comparison to the active phase of VL (Fig 2A–2D). Interestingly, IgG3 had a most expressive significant decrease 1mpt (p <0.0001), and the levels were similar to the healthy controls (median: 0.9, IQR: 0.5–1.5) in the majority of VL patients (Fig 2C).

**Fig 3:**
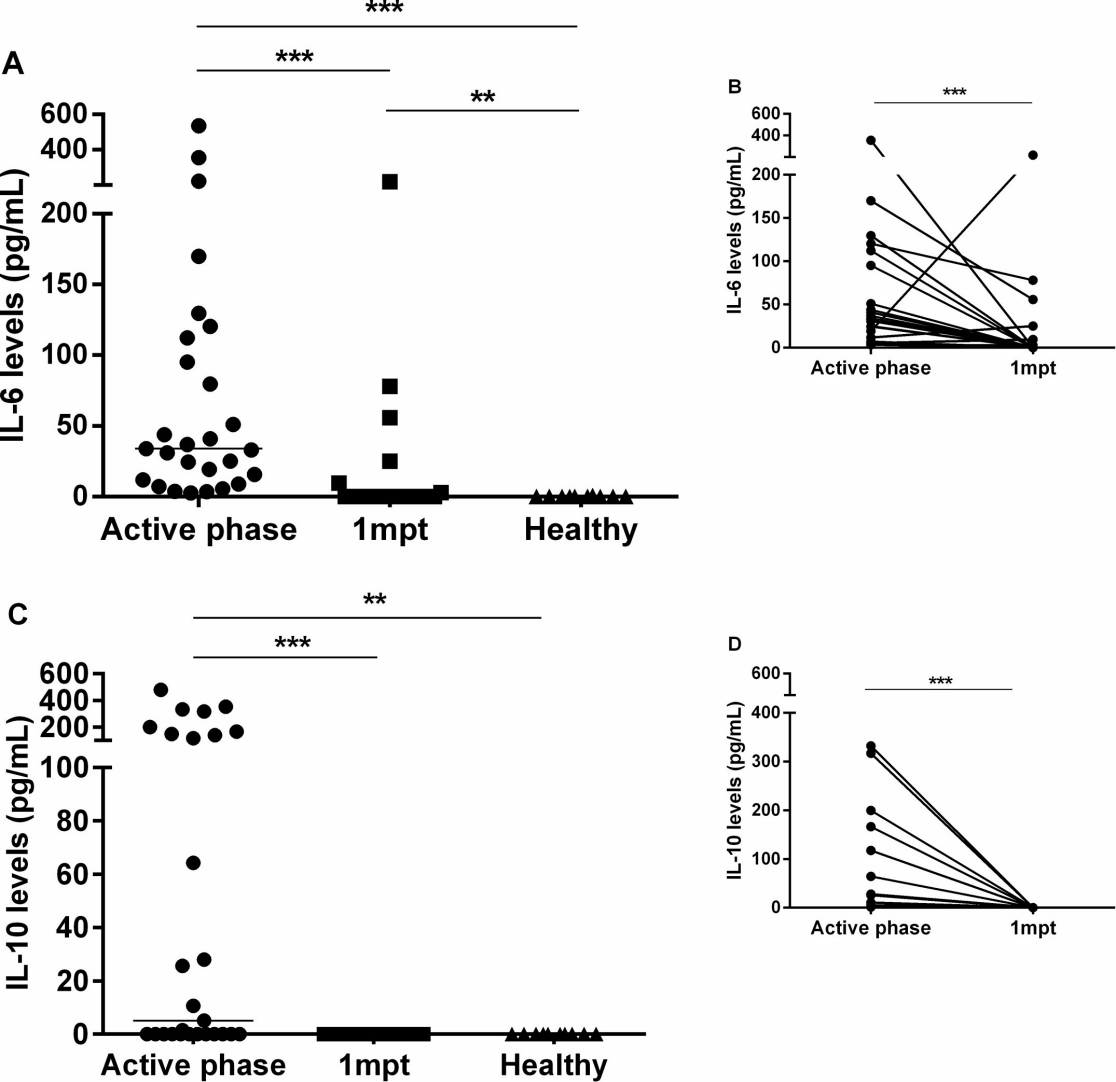
Plasma IL-6 and IL-10 levels in visceral leishmaniasis (VL) patients during clinical follow up. **(A)** IL-6 and **(C)** IL-10 levels were analyzed in the active phase and one-month post-treatment (1 mpt) in VL patients and in healthy volunteers. **(B and D)** The insert shows the IL-6 and IL-10 levels from the same patients prospectively evaluated in two different time points (n = 22). Each symbol represents one patient. The horizontal bar represents the median values. Asterisks denote a statistically significant difference between groups, *p<0.05; **p<0.01.

Consistent with VL immunopathogenesis, active VL patients presented higher levels of IL-6 (IL-6 median: 33.8 pg/mL, IQR: 11.9–112.3 pg/mL) and IL-10 (median: 5.11 pg/mL, IQR: 0.1–148 pg/mL) when compared to the post-treatment levels (IL-6 median: 0.12 pg/mL, IQR: 0.11–6.2 pg/mL, IL-10 median: 0.10 pg/mL, IQR: 0.10–0.12 pg/mL) (Fig 3A–3D).

### 3.4 Plasma leptin levels are increased in VL patients after anti-*Leishmania* treatment

**Fig 4:**
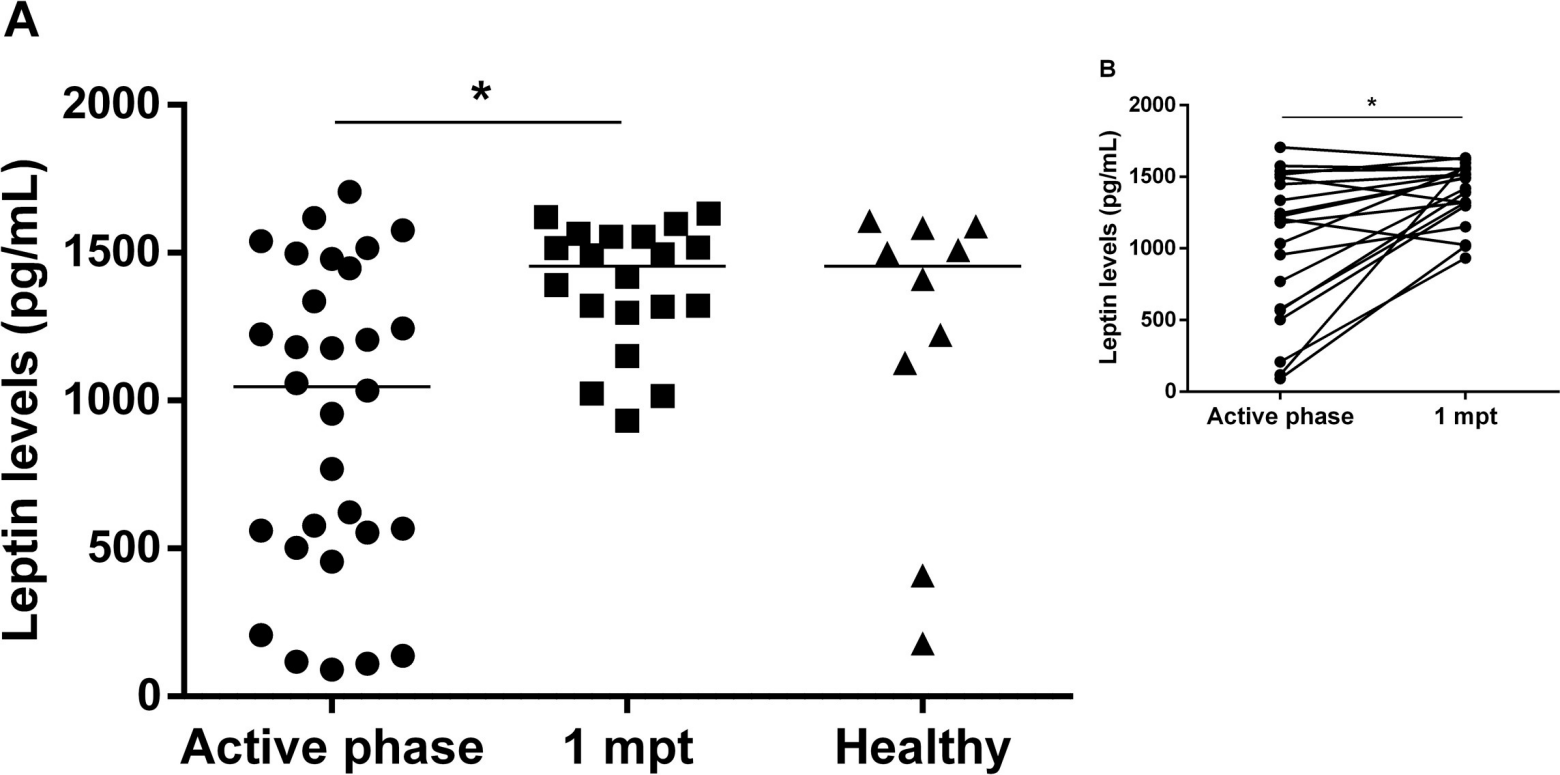
Levels of plasma leptin in patients with visceral leishmaniasis during clinical follow up (VL). **(A)** Leptin levels of VL patients in the active phase (n = 31), after one-month post-treatment (1 mpt, n = 22) and in healthy volunteers. **(B)** The insert shows the same patients prospectively evaluated in two different time points (n = 22). Each symbol represents one patient. The horizontal bar represents the median values. Asterisks denote a statistically significant difference between groups, *p<0.05; **p<0.01.

Considering that leptin is a pleiotropic protein related to metabolism and immune function, we investigated whether the plasma leptin levels are affected in VL. During the active phase of VL, patients (median: 1,047 pg/mL; IQR: 541–1,455 pg/mL) showed lower levels of plasma leptin compared to healthy controls (median: 1,455 pg/mL; IQR: 948–1,585 pg/mL) (Fig 4A). On the other hand, a significant augment of these levels after one-month post-treatment was observed (median: 1,455 pg/mL; IQR: 1,302–1,555 pg/mL) (Fig 4A and 4B), reaching similar levels to those presented by healthy controls. It was interesting to note that as observed with LPS, IgG, and cytokines, leptin levels were also dispersed in the active phase of VL patients. We then decided to investigate the possible association of these biomarkers with VL severity-associated laboratory parameters.

**Fig 5:**
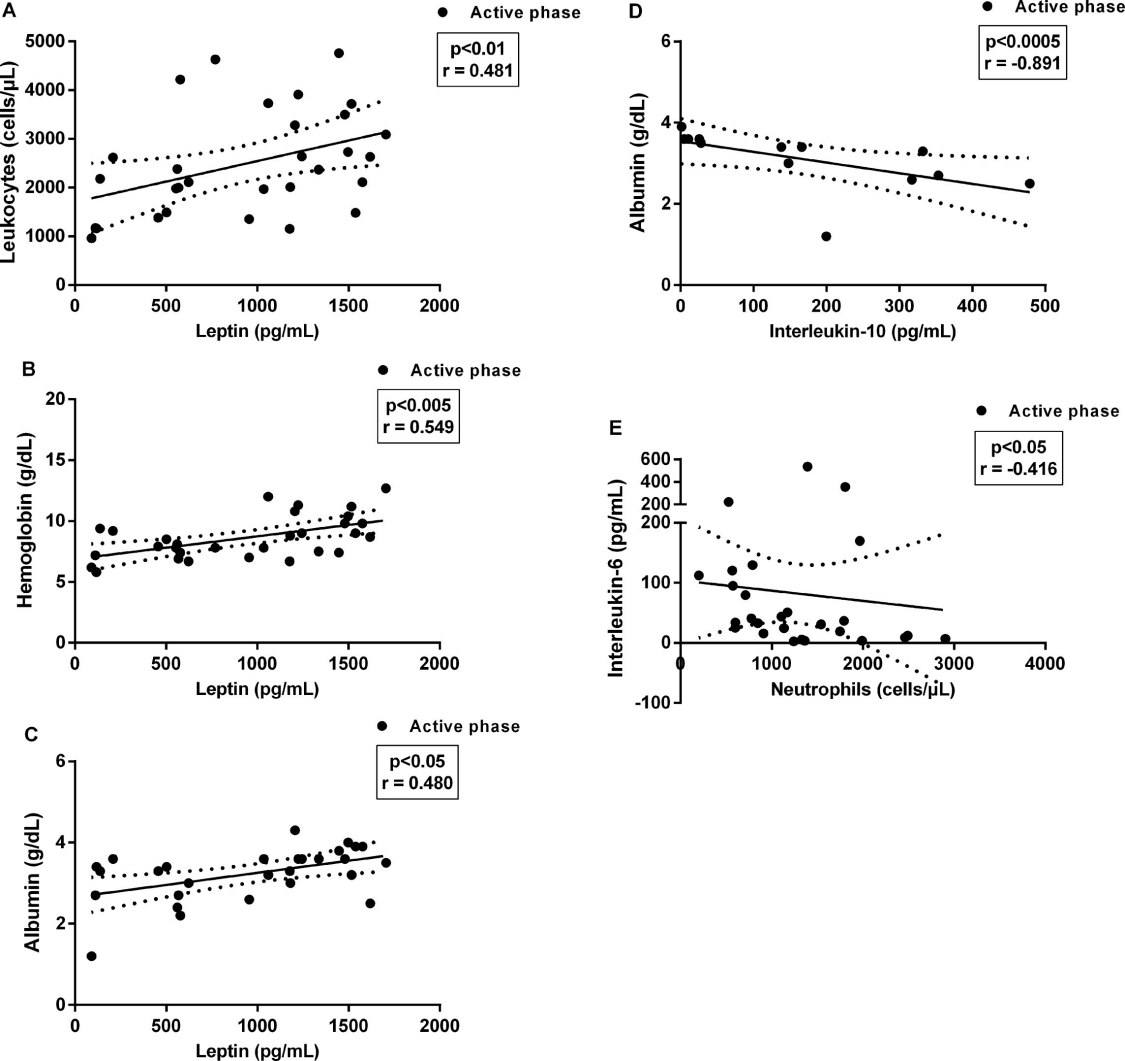
Correlations between laboratorial biomarkers of severity and leptin and cytokine levels in visceral leishmaniasis (VL) patients during active phase. Positive correlations between the leptin levels and **(A)** leukocytes, **(B)** hemoglobin and **(C)** albumin levels of the VL patients in the active phase of disease. Negative correlations between IL-10 and IL-6 levels and albumin and neutrophils levels **(D and E, respectively)**. Each symbol represents one patient in the active phase of VL. Spearman correlation. p<0.05, p<0.01, p<0.005.

In this context, only leptin levels were positively correlated with lower leukocytes, hemoglobin, and albumin levels (r = 0.48, r = 0.55, and r = 0.48, respectively) (Fig 5A–5C), indicating that reduction of leptin is also related to VL severity. On the other hand, negative correlations were verified between albumin and IL-10 levels (r = -0.89) (Fig 5D) and between neutrophils and IL-6 levels (r = –0.42) (Fig 5E).

## 4. Discussion

Systemic inflammatory response syndrome (SIRS) is the immunopathogenic substrate of several diseases such as sepsis [20], severe malaria [21], dengue [22], more recently, ebola [23] and severe influenza [24], and also visceral leishmaniasis [3] in which there are strong correlations between the abrupt release of multiple cytokines by the immune system (cytokine storm) and laboratory and clinical parameters

In order to understand the dynamics of immunological restoration, we monitored the kinetics of soluble immune mediators before and after specific therapy. As expected, elevated levels of LPS and IgG1 and G3 anti-*Leishmania* were observed in comparison to healthy individuals in addition to plasmatic cytokines known to be increased in this phase of disease [8–10], particularly IL-6 and -10. Such an increase in IL-levels are consistent with the activation degree observed in VL patients in the active phase [7, 25].

We have shown for the first time that LPS could have a role in the pathogenesis of VL patients, since it was correlated with T-cell activation, macrophage activation, and pro-inflammatory cytokine such as IL-6 and -8 and MIF [7]. Since we only evaluated a few patients before and at different times post-therapy, we decided now to investigate a larger cohort at one month after therapy. In accordance with previous data, LPS levels were significantly higher in the active phase of disease in comparison to healthy individuals and slightly decreased after one-month post-treatment. However, even the patients evaluated six months after therapy did not present normal LPS levels [7]. Thus, it seems that despite a successful response to the specific therapy, the heightened cellular activation status takes time to return to immune homeostatic status.

The origin of LPS is still unclear, but there is evidence that it comes from Gram negative bacteria in the intestinal lumen. In accordance with this idea, diarrhea is one of the most frequent symptoms of VL patients, and the presence of *Leishmania* has been demonstrated in intestinal tissue [26,27] since the time of the first cases of the disease. It is important not to confound this supposed Gram-negative bacteremia with the infection caused by Gram-positive bacteria, which is a clinical condition frequently described in VL patients [3]. Thus, our results support that the higher plasmatic LPS levels in VL patients is not associated with bacterial co-infection but rather with the possibility of microbial translocation from lumen into the blood circulation, which in turn may contribute to worsening of VL patients’ immunological status. Recently, in corroboration with our results, sCD14 levels, a soluble receptor of LPS, also correlated with inflammatory cytokine levels [9]. Altogether, these results reinforce that both LPS and parasitic antigens may trigger these inflammatory mechanisms [7].

IL-6 is elevated in active VL and is also associated with severity parameters (edema, vomiting, and bleeding) and lethality [8, 10]. The current results highlight once more the importance of IL-6 as a key cytokine in the VL pathogenesis. Even though IL-6 was elevated in active VL, the levels were not associated with laboratory parameters of severity and even declined after therapy. It is in agreement with the clinical status of our patients once they did not fulfill criteria for poor prognosis and evolved with a good clinical outcome. However, it was observed a negative correlation between IL-6 levels and neutrophil count, which can reinforce that neutropenia, thrombocytopenia and anemia are not only a consequence of depletion of bone marrow cellularity by *Leishmania* infiltration but also, a consequence of systemic inflammation [8]. Further studies with experimental models should inform us as to whether immunobiological molecular targeting for neutralizing IL-6 effects, along with anti-*Leishmania* therapy, could contribute to diminishing immune activation [28]. Finally, albumin was negatively correlated to IL-10 levels. Although there is no direct relation between them, both are parameters known to be associated with severity of VL, since high levels of IL-10 in the active phase of the disease may contribute to parasite proliferation and a successful infection. At the same time, low levels of albumin in active LV, along with plasma leakage may contribute to edema, another important parameter of severity.

More recently, leptin that is produced and secreted by several tissues, especially adipose tissue, was increased and was able to predict poor outcomes in sepsis patients [29]. Meanwhile, with respect to *L*. *donovani* infection, *in vitro* studies with recombinant leptin caused an increase in microbicidal activity of infected macrophages of both THP-1 lineage or human cells via enhancement of intracellular ROS generation [17]. On the other hand, its deficiency may worsen VL pathogenesis for contributing to Th2 polarization and impaired phagocytic activity. In addition, leptin restored the cellular immunity of mice infected with *L*. *donovani* contributing to the decrease in the parasitic burden through inhibition of arginase activity and regulation of expressions of molecules related to cellular exhaustion (such as CTLA-4/CD152 and programmed cell death protein (PD)-1 [30, 31].

To our knowledge, the current study is the first report concerning assessment of leptin levels in human VL from a prospective view. Our results indicate that leptin is altered in human VL once patients present diminished plasma leptin levels during the active phase of disease. It is important to note that leptin levels were not affected in around 50% of patients. Moreover, those presenting lower leptin levels in the active VL phase increased the leptin levels post anti-*Leishmania* therapy and reached similar values to those observed in healthy individuals. Although the patients included in this study were clinically negative for bacterial infection, assessment of leptin levels in serum alone could not be enough to establish their involvement in the disease. However, in the present study, it is clear that leptin levels are affected in VL patients during active phase of disease and these levels were positively correlated with leukocytes counts and hemoglobin and albumin levels, which are important parameters associated with VL prognosis. Altogether, our data suggest that in fact such a molecule may be related to several immune functions. Future studies are needed to clarify whether such alteration is directly caused by *L*. *infantum* infection or some consequence of LV.

One can consider that the leptin decrease is a consequence of weight loss since it is produced in amount proportional to body fat. However, in this study, we could not address this point since it was difficult for a patient to inform us of the number of pounds he/she lost since the onset of the disease. Despite identification of leptin as a hallmark biomarker in malnutrition, a previous study also verified a drastic fall in the serum leptin levels in *L*. *donovani* infected mice fed with a normal diet [17], confirming that the circulating leptin levels might be downregulated during *Leishmania* infection. It remains to be evaluated whether the malnutrition prior to or even the weight loss during the course of active VL potentiates the decrease of serum levels of leptin.

There was no correlation between leptin and immunological parameters, probably due to the fact that the patients did not progress to severe VL. In addition, in the current study all of the enrolled patients were adults <50 years old, which was different from what has been described in the literature with children or individuals >50 years old [8, 9, 32].

In conclusion, as far as we know, this work is the first to show that leptin production is affected in patients with VL. As suggested by experimental studies, the decrease in leptin levels can impair macrophage function and consequently increase the parasitic burden. The association between lower leptin levels and lower leukocyte counts raises the hypothesis of this molecule’s involvement in VL immunopathogenesis. Keeping in mind that administration of recombinant leptin favored the decrease of *Leishmania* infection in infected mice [30], we can argue that recovery of leptin levels after therapy can be related to a restored effector immune response. Taken together, our results suggest that leptin may be potentially used as a prognostic marker of VL and indicates that further studies are needed to assess the real role of leptin in the immune responses in VL.

## Abbreviations

VL: visceral leishmaniasis
1mptto: one-month post treatment
